# Heart rate variability based on risk stratification for type 2 *diabetes mellitus*

**DOI:** 10.1590/S1679-45082017AO3888

**Published:** 2017

**Authors:** Julia Silva-e-Oliveira, Pâmela Marina Amélio, Isabela Lopes Laguardia Abranches, Dênis Derly Damasceno, Fabianne Furtado

**Affiliations:** 1 Instituto Federal do Sudeste de Minas Gerais, Barbacena, MG, Brazil.

**Keywords:** Autonomic nervous system, Diabetes mellitus, Heart rate, Risk, Cardiovascular diseases

## Abstract

**Objective:**

To evaluate heart rate variability among adults with different risk levels for type 2 *diabetes mellitus*.

**Methods:**

The risk for type 2 *diabetes mellitus* was assessed in 130 participants (89 females) based on the questionnaire Finnish Diabetes Risk Score and was classified as low risk (n=26), slightly elevated risk (n=41), moderate risk (n=27) and high risk (n=32). To measure heart rate variability, a heart-rate monitor Polar S810i^®^ was employed to obtain RR series for each individual, at rest, for 5 minutes, followed by analysis of linear and nonlinear indexes.

**Results:**

The groups at higher risk of type 2 *diabetes mellitus* had significantly lower linear and nonlinear heart rate variability indexes.

**Conclusion:**

The individuals at high risk for type 2 *diabetes mellitus* have lower heart rate variability.

## RESUMO

**Objetivo:**

Avaliar a variabilidade da frequência cardíaca em adultos com diferentes níveis de risco para *diabetes mellitus* tipo 2.

**Métodos:**

O grau de risco para *diabetes mellitus* tipo 2 de 130 participantes (41 homens) foi avaliado pelo questionário *Finnish Diabetes Risk Score*. Os participantes foram classificados em baixo risco (n=26), risco levemente elevado (n=41), risco moderado (n=27) e alto risco (n=32). Para medir a variabilidade da frequência cardíaca, utilizou-se o frequencímetro Polar S810i^®^ para obter séries de intervalo RR para cada indivíduo, em repouso, durante 5 minutos; posteriormente, realizou-se análise por meio de índices lineares e não-lineares.

**Resultados:**

O grupo com maior risco para *diabetes mellitus* tipo 2 teve uma diminuição significante nos índices lineares e não-lineares da variabilidade da frequência cardíaca.

**Conclusão:**

Os resultados apontam que indivíduos com risco alto para *diabetes mellitus* tipo 2 tem menor variabilidade da frequência cardíaca.

## INTRODUCTION

The incidence of *diabetes mellitus* (DM) has increased worldwide at epidemic proportions, especially among older, less active and/or more obese individuals.^1^ The International Diabetes Federation estimates that 336 million people have been diagnosed as DM, and approximately 4.6 million diabetes-related deaths occur every year.^2^ The future scenario does not look better. According to forecasts, DM will have an increasing impact on years of life lost due to premature death and disability, all over the world, moving from the 11^th^ to 7^th^ cause of death by 2030.^3^

Type 2 DM (T2DM) individuals present reduced autonomic function in cardiovascular system,^4^ evidenced by decreased heart rate variability (HRV), which results in cardiac autonomic neuropathy and increased risk for sudden cardiac death.^5,6^ HRV is a non-invasive measurement that indirectly reflects the cardiac autonomic regulation. Its analysis is based on fluctuations in the sequential RR intervals from sinus rhythm.^7,8^

The Finnish Diabetes Risk Score (FINDRISC) is a data collection tool validated by the Department of Public Health of the University of Helsinki, Finland.^9^ The original instrument, composed of eight questions, was adapted by the Ministry of Health to the Brazilian reality. It establishes five risk categories: low, slightly elevated, moderate, high and very high.

Penčić-Popović et al.^8^ were the pioneers in showing that the group classified as slightly elevated risk for T2DM (score 12; FINDRISC≥7; n=39) already had changes in linear HRV parameters (time and frequency domain) when compared to the low risk group (FINDRISC<7; n=30). These authors did not include individuals with moderate, high and very high risk, or analysis of nonlinear methods.

The time and frequency domain parameters of HRV may not represent the non-stationary characteristics of electrocardiography (ECG) due to the nonlinear dynamics of heart rhythm.^10^ The nonlinear methods, such as Poincaré plot^11^ and tone-entropy analysis,^12^are newly developed tools to identify nonlinear patterns of ECG data.^10^

## OBJECTIVE

To evaluate heart rate variability, among adults with different risk levels for type 2 *diabetes mellitus.*

## METHODS

This is an analytical cross-sectional study carried out from November 2014 to April 2015, after approval by the Research Ethics Committee of the *Instituto Federal do Sudeste de Minas Gerais*, Brazil, under protocol number 822.457 and CAAE: 31828814.0.0000.5588.

The study enrolled 130 individuals, mean age of 51.0 years (range of 35-70 years; standard deviation − SD 10.0 years) with no diagnosis of T2DM, and presenting severe arterial hypertension and/or heart disease. The Finnish Diabetes Risk Score was applied to assess the risk of developing T2DM within 10 years. This questionnaire addresses age, body mass index (BMI), waist circumference, physical activity, daily intake of fruits and/or vegetables, use of antihypertensive drugs, previous history of glycemic values and family history of diabetes. This tool allows a maximum score of 26 points, and scores higher than 15 indicate high probability of developing T2DM. Participants were allocated to four groups: Group 1 for those who scored <7 points and had low risk (n=26); Group 2 for those who scored from 7 to 11 points and presented slightly elevated risk (n=41); Group 3 for those who scored from 12 to 14 points and showed moderate risk (n=27) and Group 4 for those who scored from 15 to 20 points and had high risk (n=32). Individuals who used antidiabetic drugs or had been diagnosed as type 1 DM were excluded.

### Individual characteristics of participants

The heart rate was extracted from data generated in the Polar Electro Oy heart rate monitor, using the Polar ProTrainer software and considering the mean, maximum and minimum values.

Systolic blood pressure (SBP) and diastolic blood pressure (DBP) were measured using a mercury-column sphygmomanometer and a stethoscope. The mean arterial pressure (MAP) was obtained based on the mathematical formula (SBP minus DBP) divided by 3+DBP.

To assess fasting glucose levels, an Accu-Check^®^ Active glucometer with Accu-Check^®^Active strips was used. Participants were oriented to fast for 8 hours and to not to drink alcoholic beverages in the previous 24 hours.

The body mass (kg) was assessed using mechanical scales, with a maximum capacity of 300kg and accuracy of 200g. Height was measured with a stadiometer coupled to the scales. The BMI was obtained by weight (kg) divided by square height (m^2^). Waist circumference (cm) was considered the smallest perimeter of the abdomen, while hip circumference (cm) was measured at the level of maximum posterior extension of the buttocks. The waist-hip ratio (WHR) was calculated by dividing the waist circumference (cm) by hip circumference (cm). To determine the percentage of fat, four-pole electrical bioimpedance analysis was performed using Biodynamics^®^ BIA 450, and the participants were on supine position. They were oriented to urinate before the test.

RR intervals for HRV assessment were obtained and recorded using the heart rate monitor Polar S810i (Polar Electro In., Finland), which was previously validated for beat-to-beat capture.^13^ The participants were on supine position and asked to remain quiet during capture. The test lasted approximately 5 minutes, as described by Ziegler et al.^14^

The data were extracted and converted into text by using the Polar ProTrainer software. The initial and final records were disregarded so that only 300 heartbeats were analyzed by HRV analysis software.^15^

HRV was analyzed according to linear (time and frequency domain) and nonlinear methods. Time domain measurements included mean RR interval (ms); standard deviation of the NN intervals (SDNN) recorded in a period of time expressed as ms; root mean squared successive differences (RMSSD) between adjacent normal RR intervals in a time interval, also expressed as ms; percentage of adjacent RR intervals differing by >50ms (pNN50); and the triangular index.

The frequency domain covers the very low frequency component (VLF), in ms^2^ and percentage, the low frequency (LF) component, in ms^2^ and percentage, the high-frequency component (HF), in ms^2^ and percentage, and the LF/HF ratio. The HF component acts in HRV, reflecting parasympathetic modulation, while the LF component includes sympathetic and parasympathetic function, and the VLF reflects parasympathetic and neuroendocrine modulation.^8^

Nonlinear methods comprised the variables short-term variability (SD1), in ms, long-term variability (SD2), in ms, approximate entropy (ApEn) and sample entropy (SampEn). The Task Force of the European Society of Cardiology and the North American Society of Pacing and Electrophysiology present detailed explanation about each variable.^16^

### Statistical analysis

Quantitative variables were presented as mean and SD and compared with FINDRISC risk groups, according to Analysis of Variance (ANOVA) and *post hoc* Tukey tests. Categorical variable (sex) was described as absolute frequency and relative frequency and was compared according to G test. Biostatisc 5.0 software was used at 5% error probability (p<0.05).

## RESULTS

Descriptive and inferential statistic values for sociodemographic and anthropometric variables, body composition and glucose levels of participants are shown in table 1. Group 4 was significantly older than Group 1 and had greater BMI, larger waist circumference and waist-hip ratio than Groups 1 and 2. The only difference between Groups 3 and 4 was body mass: Group 4 exceeded Group 3 by approximately 10kg. There were no differences between Groups 1 and 2 for the variables shown in table 1. Fasting glucose was slightly lower (p=0.05) in the Group 1.

**Table 1:** Characterization of the groups by quantitative and qualitative variables according to the Finnish Diabetes Risk Score questionnaire

Variables	Group 1	Group 2	Group 3	Group 4	p value	Multiple comparisons between groups*
	(n=26)	(n=41)	(n=27)	(n=32)		
Age, years^†^	46.5 (7.5)	51.4 (8.6)	52.1 (7.2)	54.5 (8.3)	0.02^‡^	1 and 4
Sex, male^§^	8 (30.7)	17 (41.5)	5 (18.5)	11 (34.4)	0.24^•^	
Body mass, kg^†^	64.4 (9.0)	66.1 (10.8)	69.2 (10.0)	78.5 (11.8)	0.0003^‡^	1 and 4; 2 and 4; 3 and 4
Height, m^†^	1.60 (0.1)	1.60 (10.8)	1.60 (0.1)	1.60 (0.1)	0.19^‡^	
BMI, kg/m^2†^	23.5 (4.7)	25.5 (3.1)	27.8 (3.0)	30.6 (4.0)	<0.0001^‡^	1 and 3; 1 and 4; 2 and 4
Waist circumference, cm^†^	79.6 (5.1)	82.8 (8.1)	87.5 (7.5)	93.9 (9.5)	<0.0001^‡^	1 and 3; 1 and 4; 2 and 4
Hip circumference, cm^†^	97.7 (4.7)	100.6 (8.0)	101.9 (5.5)	105.7 (8.0)	0.01^‡^	1 and 4
WHR^†^	0.8 (0.0)	0.8 (0.1)	0.9 (0.1)	0.9 (0.1)	0.001^‡^	1 and 4; 2 and 4
Percentagen of fat^†^	32.5 (11.5)	33.3 (5.1)	35.5 (3.8)	36.4 (5.7)	0.0004^‡^	1 and 3; 1 and 4
Fasting glucose	89 (4.9)	99.2 (13.2)	100.2 (11.6)	102.3 (13.8)	0.05^‡^	
SBP, mmHg	119.2 (8.6)	119.6 (8.3)	119.1 (11.9)	121.1 (10.1)	0.92^‡^	
DBP, mmHg	77.3 (6.6)	76.6 (8.2)	79.3 (7.7)	78.9 (8.2)	0.66^‡^	
MAP, mmHg	90.4 (10.5)	92.6 (7.4)	92.3 (10.5)	93.7 (7.9)	0.76^‡^	
Minimum HR, bpm	60.8 (5.6)	61.5 (7.7)	64.2 (9.0)	61.5 (7.0)	0.46^‡^	
Mean HR, bpm	67.6 (5.5)	68.0 (7.5)	68.7 (11.3)	67.7 (6.8)	0.97^‡^	
Maximum HR, bpm	78.8 (6.9)	78.7 (9.7)	80.4 (9.9)	75.9 (7.4)	0.54^‡^	

*: Tukey test; ^†^: data described as mean (standard deviation); ^‡^: Analysis of Variance; ^•^G-test; ^§^data described in absolute values (relative).

BMI: body mass index; WHR: waist-hip ratio; SBP: systolic blood pressure; DBP: diastolic blood pressure; MAP: mean arterial pressure; HR: heart rate; bpm: beats per minute.

By and large, the cardiovascular variables SBP, DBP and MAP as well as maximum, minimum and mean heart rate, had no significant difference among groups.

Table 2 describes the results for time domain. The variables SDNN (p=0.04), RMSSD (p=0.007), pNN50 (p=0.004) and triangular index (p=0.01) were significantly lower in the groups with higher scores, indicating that these groups had lower parasympathetic activity.

**Table 2:** Heart rate variability in the time domain

Variables	Group 1	Group 2	Group 3	Group 4	p value*	Multiple comparisons between groups^†^
	(n=26)	(n=41)	(n=27)	(n=32)		
Mean RR, ms	904.7 (81.3)	911.8 (96.9)	869.7 (122.1)	915.7 (104.4)	0.53	
SDNN, ms	64.7 (19.4)	56.3 (17.8)	53.2 (22.5)	47.2 (16.0)	0.04	1 and 4
RMSSD, ms	43.0 (16.6)	39.0 (14.9)	35.7 (20.7)	25.2 (9.9)	0.007	1 and 4; 2 and 4
pNN50, %	19.4 (12.6)	17.0 (12.4)	12.3 (12.7)	6.8 (6.5)	0.004	1 and 4; 2 and 4
Triangular index	14.0 (3.3)	12.2 (3.5)	11.4 (3.7)	10.4 (2.7)	0.01	1 and 4

Data described in mean (standard deviation).

^*^: Analysis of Variance; ^†^: Tukey test.

SDNN: standard deviation of the NN intervals; RMSSD: root mean squared successive differences; pNN50: percentage of adjacent RR intervals differing by >50ms.

Results for frequency domain are shown in table 3. The components VLF (%) and LF (ms^2^ and %) were lower (p=0.0002, p=0.003 and p=0.002, respectively) in the group with the highest score, in which HF (%) was also lower (p=0.03), indicating possible silent cardiac autonomic dysfunction. The sympathetic-vagal balance given by LF/HF ratio was not significantly different among groups.

**Table 3:** Heart rate variability in the frequency domain

Variables	Group 1	Group 2	Group 3	Group 4	p value*	Multiple comparisons between groups^†^
	(n=26)	(n=41)	(n=27)	(n=32)		
VLF						
ms^2^	2,193.3 (1700.7)	1,658.5 (1198.2)	1,554.9 (1321.5)	1,537.0 (1084.5)	0.55	
%	47.5 (14.6)	48.5 (14.6)	51.4 (12.1)	64.4 (10.3)	0.0002	1 and 4; 2 and 4; 3 and 4
LF						
ms^2^	1,519.4 (993.7)	1,044.5 (652.1)	1,003.9 (887.2)	526.9 (360.1)	0.003	1 and 4
%	36.9 (11.3)	33.2 (12.0)	33.9 (12.3)	24.1 (7.9)	0.002	1 and 3; 2 and 4; 3 and 4
HF						
ms^2^	608.7 (490.5)	581.4 (452.2)	576.0 (618.1)	252.2 (189.6)	0.17	
%	15.6 (7.0)	18.2 (8.6)	14.6 (6.5)	11.5 (5.7)	0.03	2 and 4
LF/HF	2,718.7 (1769.3)	2,097.2 (1409.4)	3,187.3 (1824.2)	3,147.2 (2078.2)	0.58	

Data described in mean (standard deviation).

^*:^ Analysis of Variance; ^†:^ Tukey test.

VLF: very low frequency; LF: low frequency; HF: high frequency.

Table 4 displays the nonlinear method results. Similarly to linear indexes, the group at the highest risk of developing T2DM (Group 4) had altered HRV when compared to Groups 1 and 2, besides showing lower SD1, SD2 and sample entropy (SampEN) values.

**Table 4:** Nonlinear indexes of the heart rate variability of the different groups obtained by the Finnish Diabetes Risk Score

Variables	Group 1	Group 2	Group 3	Group 4	p value*	Multiple comparisons between groups^†^
	(n=26)	(n=41)	(n=27)	(n=32)		
SD1, ms	30.5 (11.7)	27.6 (10.6)	25.3 (14.7)	17.9 (7.0)	0.007	1 and 4; 2 and 4
SD2, ms	85.9 (25.7)	74.2 (23.4)	70.3 (28.1)	63.9 (21.7)	0.05	
ApEn	1.0 (0.1)	1.0 (0.1)	1.0 (0.1)	1.0 (0.1)	0.16	
SampEn	1.5 (0.2)	1.5 (0.2)	1.4 (0.3)	1.3 (0.2)	0.008	2 and 4

Data described as mean (standard deviation).

^*^: Analysis of Variance; ^†^: Tukey test^.^

SD1: short-term variability; SD2: long-term variability; ApEn: approximate entropy; SampEn: sample entropy.

## DISCUSSION

Previous studies reported decreased HRV for diabetic patients with or without associated diabetic neuropathy.^17-20^ To date, only one study has correlated FINDRISC risk of developing T2DM with HRV measurements.^8^Penčić-Popović et al.^8^analyzed only two groups − low risk (score <7) and slightly elevated risk (score 7-11) −, based on linear indexes. Therefore, this is the first study to include the moderate risk (score 12 to 14) and the high risk (score 15 to 20) groups in the HRV analysis based on linear and nonlinear indexes. The methods used were different, since in the study by Penčić-Popovic et al.,^8^ the ECG was used for 24 hours, while in the present study a Polar S810i was used for 5 minutes, at rest. These measurements at rest correlate and agree with the circadian variation described in the ECG.^21^

The groups that obtained higher scores, especially Group 4, showed lower values of both parasympathetic (RMSSD, pNN50 and HF) and sympathetic modulation (LF), resulting in lower values of SDNN, triangular index and nonlinear indexes (SD1, SD2 and sample entropy). Different degrees of glucose intolerance are inversely proportional to reduced HRV.^14^ The major reasons for autonomic cardiovascular dysfunction were found in individuals who had lower glucose tolerance, newly diagnosed diabetes and diabetes, respectively. Penčić-Popović et al.^8^ observed compromised sympathetic and vagal modulation in the slightly elevated risk group (7≤ FINDRISC <12), due to decrease in SDANN (p=0.035), VLF (p=0.03), LF (p=0.006) and HF (p=0.011) values, compared to the low-risk group (score <7). Some studies^20,21^ found significantly decreased SDNN, HF and LF in diabetic patients and in individuals with high blood glucose, and the reduced HRV was identified as a sensitive marker for subclinical autonomic neuropathy.^19^

Adopting only parameters in the time and frequency domain to analyze HRV decrease is not always suitable due to variance in data and presence of nonlinear phenomena.^22^ The graphic analysis according to Poincaré plot and sample and approximate entropy have been increasingly used because they compute shorter HRV intervals and provide additional information about each RR interval, which cannot be obtained by means of conventional analysis methods.^22^ In this study, there was a reduction in entropy for Group 4, compared to Groups 1 and 2, considering the sample entropy parameters (p=0.08), SD1 (p=0.007) and SD2 (p=0.05). Similarly, in a study^23^ with 63 patients showing T2DM and 29 clinically healthy individuals, approximate entropy was not significantly different (p=0.199); however, the curve for healthy participants was higher than for diabetics, suggesting that the decrease in entropy is correlated with compromised vagal modulation on the heart. Since decreased circadian rhythm values have been strongly associated with mortality, the individuals with higher FINDRISC scores should be more often monitored for cardiovascular risk factors.^19^

The hemodynamic variables (heart rate and BP) did not vary among groups. However, there is evidence that the increase in heart rate is proportional to the rise in blood glucose levels in individuals with no diagnosis of T2DM, especially when compared to diabetic patients.^19,24^ In addition, increased SBP is considered a feature of DM2^25^ and is correlated with increased FINDRISC scores for both sexes.^18^ The increase by 20mmHg in SBP or 10mmHg in DBP doubles the risk of death from heart attack, ischemia and other vascular complications.^26^The use of antihypertensive drugs has been associated with increased risk of T2DM^26^ and was not discontinued for HRV tests.

The anthropometric measurements (BMI and waist circumference) found for participants in this study were lower than results reported by Penčić-Popović et al.,^8^ Makrilakis et al.^1^and Lindström et al.,^9^ who also used the FINDRISC. In the present study, the majority of the study population had waist circumference inferior to the cutoff values for cardiovascular disease risk (88cm for women and 102cm for men). However, the higher the FINDRISC, the higher the percentage of abdominal fat and adiposity; thus, waist circumference can act as a good tool to predict visceral obesity, according Meijnikman et al.^27^

The gradual decrease in HRV parameters (both in time domain and in frequency domain) correlated with increased age, BMI and visceral fat, has been associated with a possible silent dysautonomia in individuals not diagnosed as DM, but with strong risk factors.^17^ In clinical practice, the use of FINDRISC as a tool for cardiovascular risk monitoring should be encouraged, considering the score was capable of identifying individuals who had compromised sympathetic and vagal modulation of the heart, even with no diagnosis of T2DM.

Thus, it must be considered that advanced age,^28^ overweight^29,30^ and abdominal obesity^31^ are factors that decrease the HRV − either associated or not with diagnosed T2DM.^17^ However, the risk of developing T2DM is known to be higher in individuals aged over 45 years and overweight, indicating that these two conditions are almost inseparable. In addition, all these items (age, BMI, abdominal circumference) are considered in the FINDRISC score. The cross-sectional design of the present study did not allow to conclude that the changes in HRV found in the comparison among groups were due to the higher risk of developing T2DM, or to these individual characteristics (age and body composition). A prospective cohort study should be conducted to elucidate this issue, as carried out by Wulsin et al.,^32^ who predicted T2DM from HRV. The analysis of HRV in the time and frequency domain using nonlinear indexes, as a function of T2DM risk stratification according to FINDRISC, based on the study by Penčić-Popović et al.,^8^ can support future cohort studies. Could there be any differentiation in predicting T2DM from HRV categorized according to FINDRISC? Could this lead to the proposal of a new FINDRISC cutoff by means of HRV analysis?

Early diagnosis provides interventions in pre-diabetes patients, which is important in the primary prevention of T2DM and its chronic complications, such as cardiac abnormalities.^27^The combination between reduced HRV and risk factors for diabetes, according to FINDRISC, may undergo changes due to modifications in lifestyle. However, T2DM individuals should be constantly and properly instructed by health professionals to adopt healthy lifestyle habits.

In the diabetes prevention program developed by Carnethon et al.,^33^ the interventions in lifestyle of individuals with altered fasting glucose or glucose intolerance resulted in decreased HR and increased HRV. In a study by Howorka et al.,^34^ HRV increased in diabetic patients who had trained on a stationary bike for 12 weeks. This result was found for patients without cardiovascular autonomic neuropathy or with the disease in its initial phase. Nevertheless, no effect on HRV was observed in patients with severe cardiovascular autonomic neuropathy. Ziegler et al.^14^ proposed that the reduction in HRV could be used as single-scale screening of cardiac autonomic dysfunction in individuals with varying degrees of glucose intolerance.

The association of FINDRISC with HRV data is an efficient method for screening the risk of developing T2DM and heart dysautonomia, which is one of its serious complications. Hence, screening for early detection of diabetes and possible complications is justified, so that the intervention will be possible and effective.

Although the present study innovates in showing changes between linear and nonlinear HRV indexes for individuals belonging to different FINDRISC risk groups for T2DM, some limitations must be pointed out. The first limitation was the small number of participants who obtained a score of >20 points (very high risk group), which prevented their inclusion in statistical comparisons. The second limitation was no differentiation between smokers and non-smokers among participants. All these gaps offer prospects for further studies.

## CONCLUSION

The groups with the highest Finnish Diabetes Risk Score had lower heart rate variability, including the variables of linear (standard deviation of the NN intervals, root mean squared successive differences, percentage of adjacent RR intervals differing by >50ms, triangular index, very low frequency component, low frequency and high frequency) and nonlinear methods (short-term variability, long-term variability and sample entropy), suggesting lower parasympathetic and sympathetic activity.
